# A case report of prolonged COVID‐19‐positive RT‐PCR for five months

**DOI:** 10.1002/ccr3.6113

**Published:** 2022-07-25

**Authors:** Zeinab Siami, Saeidreza Jamalimoghadamsiahkali, Armin Khavandegar

**Affiliations:** ^1^ Infectious Disease Department Alborz University of Medical Sciences Karaj Iran; ^2^ Department of Infectious Disease School of Medicine, Ziaeian Hospital, Tehran University of Medical Sciences Tehran Iran; ^3^ Student Research Committee Alborz University of Medical Sciences Karaj Iran

**Keywords:** COVID‐19, lymphoma, RT‐PCR

## Abstract

The COVID‐19 gold standard assessment tool remained the RT‐PCR of upper respiratory tract specimen extracted by the nasopharyngeal swab. A positive result would decrease through a three‐week course and eventually be undetectable. The maximum duration of viral shedding is 83 days. Besides, COVID‐19 RT‐PCR remained positive for 74 days in a patient suffering from lymphoma. In this study, we have presented a 56‐year‐old male patient, a known case of lymphoma since 2015, who experienced many episodes of chemotherapy with a five‐month positive RT‐PCR COVID‐19 laboratory test and finally was intubated and then died of opportunistic pulmonary infections. COVID‐19 patients with concurrent lymphoma failed to remove the virus thoroughly, despite providing appropriate treatment regimens.

## Abstract 2

Patients with underlying disorders affecting immune system competency would fail to remove viruses thoroughly, and viral shedding may last long enough to cause disastrous outcomes.

## 1 INTRODUCTION

The COVID‐19 gold standard assessment tool remains the RT‐PCR of upper respiratory tract specimens extracted by the nasopharyngeal swab. A positive result would dwindle through a three‐week course and eventually be undetectable.^1^

In patients with a lower cycle threshold and severe pattern of COVID‐19, RT‐PCR could remain positive for extended periods. In some patients, a detectable positive RT‐PCR for longer than 6 weeks was reported.^1^ SARS‐CoV‐2 RT‐PCR remained positive for 74 days in a patient with lymphoma.^2^ The maximum duration of viral shedding is 83 days.^3^

## 2 CASE PRESENTATION

**FIGURE 1:**
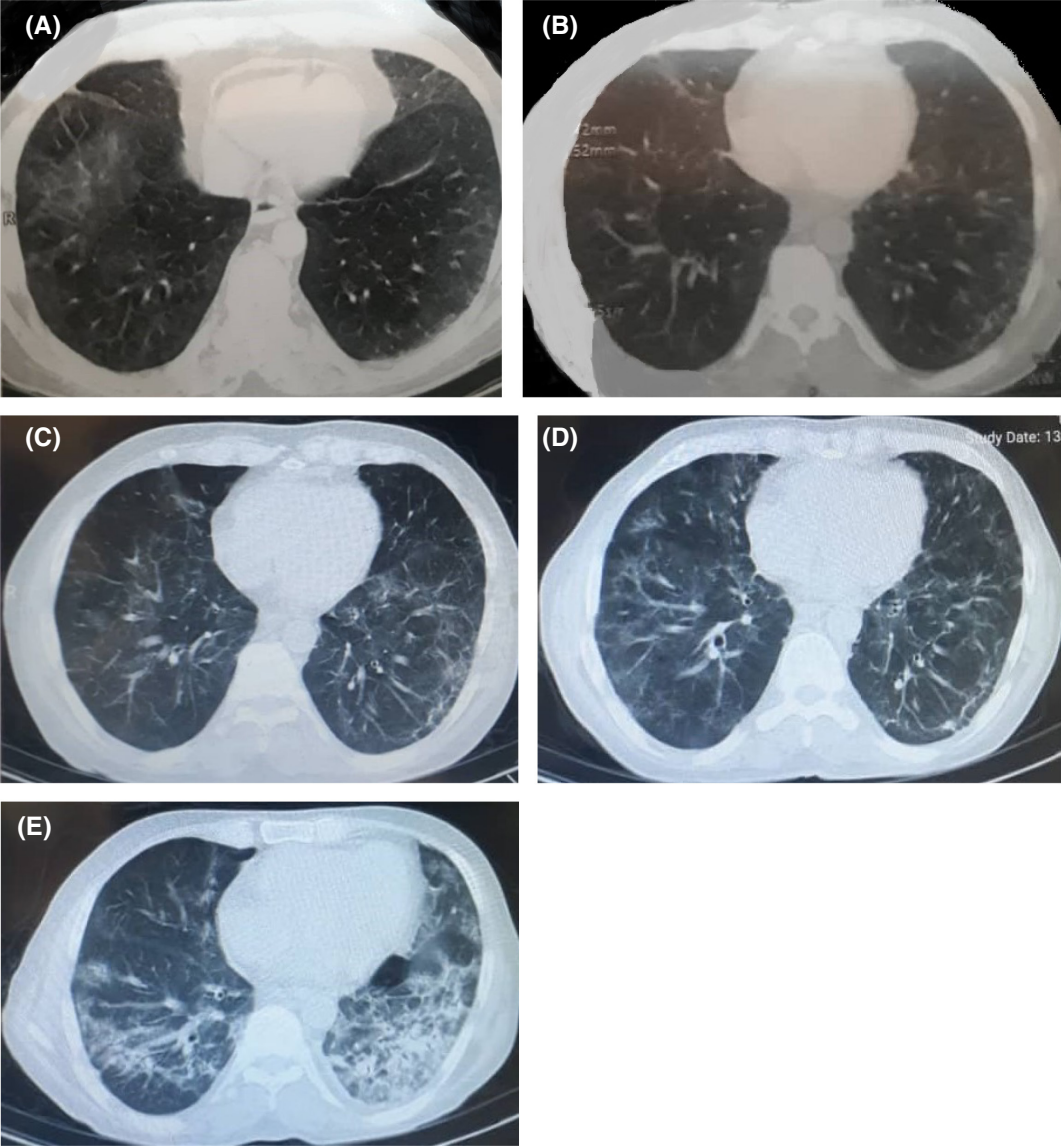
Chronological changes in CT scan

The patient is a 56‐year‐old male, a known case of lymphoma since 2015, who experienced many episodes of chemotherapy nearly every 3 weeks. The chemotherapy regimen included a combination of cyclophosphamide, doxorubicin, and vincristine. The last chemotherapy episode was on March 18, 2020. On March 23, 2020, he visited the hospital complaining of symptoms, including anorexia, fever, diarrhea, headache, and myalgia; the O2 saturation was desirable (95%), and no complaining of cough and dyspnea existed. A nasopharyngeal/oropharyngeal specimen, with the impression of COVID‐19 infection, yielded a positive result. The cycle threshold for RT‐PCR was 30. At the same time, the CT scan reported “bilateral multilobular peripherally ground‐glass opacities” (Figure 1A).

A seven‐day regimen of hydroxychloroquine was initiated. Two weeks later, on April 5, 2020, to properly decide on chemotherapy reinitiating, a nasopharyngeal specimen was extracted using a swab. With a cycling threshold of 30, the RT‐PCR yielded a positive result once more. Due to excellent general condition, O_2_ saturation of 94%, and lack of any further progression in CT findings (Figure 1b), no drug regimens were prescribed, and the patient underwent supportive treatment. Meanwhile, the patient did not receive any medication for his underlying disease. The laboratory findings revealed a positive quantitative CRP, hemoglobin = 10.9, platelet = 101,000, and normal liver and renal function tests.

On May 4, the patient visited the hospital again with the complaint of cough, fatigue, and myalgia. An RT‐PCR was requested, which yielded a positive result again. No progressive changes were reported in CT‐scan findings compared to the previous one (Figure 1c). O2 saturation was 93%. Due to the patient's stability, an outpatient five‐day regimen of interferon‐beta was initiated.

A few weeks later, on June 21, the patient visited the hospital complaining of intermittent cough and dyspnea. The CT scan demonstrated “generalized peripheral and peri‐bronchial ground‐glass opacities with increased thickness of interlobular septa” (Figure 1d). Meanwhile, the O2 saturation was 88%, which led to patient hospitalization. An RT‐PCR of nasopharyngeal secretions revealed a positive COVID‐19 result, using a cyclic threshold of 30. The physician started a combination regimen of atazanavir/ritonavir (300/100), accompanied by a corticosteroid based on the national COVID‐19 treatment guideline. Further laboratory evaluation was performed, which is depicted in Table 1.

**TABLE 1:** Patient's Laboratory findings on admission

Laboratory test	Result	Normal range
Prolactin	<0.05	<0.05 ng/mL
Ferritin	692	12–300 ng/mL
D‐dimer	0.2	<0.4mcg/ml
Alkaline Phosphatase	535	20–140 IU/L
Calcium	7.8	8.5–10.5 mg/dL
Phosphate	3.5	3.5–4.5 mg/dL
Sodium	131	135–145 mg/dL
Potassium	4.4	3.5–5.2 mmol/L
Magnesium	1.7	1.7–2.2 mg/dL
INR	0.99	<1.1
CRP	37	<10 mg/L
Creatinine	0.94	0.8–1.2 mg/dL
Cholesterol	119	<120 mg/dL
TAG	124	<150 mg/dL
AST	34	5–40 U/L
ALT	31	5–40 U/L

Despite the corticosteroid plus atazanavir treatment regimen, the patient did not reveal any remission side, and even clinical manifestations were exacerbated, including fatigue and myalgia. O_2_ saturation decreased to 85%. RT‐PCR showed a positive result even with a higher load (Cyclic threshold = 19). The corticosteroid drug used at this stage was intramuscular dexamethasone, eight milligrams daily for nearly 7 days, and then tapered gradually.

On June 28, 2020, the management team decided to start the Remdesivir regimen. After almost 7 days of treatment, on July 6, 2020, the patient's general condition was desirable and stable; a chest CT scan (Figure 1e) revealed no further progression, and the cyclic threshold increased. In this time, the laboratory findings consisted of WBC=6900, hemoglobin=11.1, platelet=70,000, FANA=0.11, and ferritine=441.26.

On July 25, 2020, the clinical condition deteriorated. The temperature was detected in the examination, and leukocytosis (WBC = 18,000 cells/mm3) and elevated CRP (86 mg/L) were revealed in laboratory findings. O_2_ saturation gradually decreased to 80%. Hence, the extended‐spectrum antibiotics and antifungal treatment regimen were initiated with the impression of bacterial and opportunistic infections empirically. All other related conditions were excluded. The blood culture for opportunistic infections remained negative; therefore, bronchoalveolar lavage using bronchoscopy was performed. The secretory secretion yielded a positive result for Streptococcus pneumonia.

Immunological investigation yielded a negative result for both IgM and IgG. Eventually, due to a lack of clinical improvement, the head of the management team decided to perform plasma therapy. After plasma therapy, no clinical improvement was observed.

Unfortunately, on August 5, 2020, the patient expired after a five‐month positive RT‐PCR. Interestingly, the RT‐PCR remained positive until the last moment. The last cycle threshold was 17. The sequence of cyclic threshold and the patient's clinical condition is summarized in Table 2.

**TABLE 2:** Sequence of cyclic threshold and patient's clinical condition

Date	Cyclic Threshold	Status	Regimen	O2 Saturation
March 23, 2020	30	outpatient	Hydroxychloroquine	95%
April 5, 2020	30	outpatient	conservative	94%
May 4, 2020	30	outpatient	Interferon‐beta	93%
June 21, 2020	30	hospitalized	Atazanavir/Ritonavir + corticosteroid	88%
June 28, 2020	19	hospitalized	Remdesivir	85%
July 6, 2020	25	hospitalized	conservative	90%
July 25, 2020	‐‐‐	ICU‐admitted	Extended spectrum antibiotics + antifungal + then plasma change	80%
August 5, 2020	17	expired		<80% (variable)

## 3 DISCUSSION

In this study, we have presented a 56‐year‐old male patient, a known case of lymphoma since 2015, who experienced many episodes of chemotherapy before getting infected with COVID‐19, and afterward, with a five‐month positive RT‐PCR COVID‐19 laboratory test who finally was intubated and then died of opportunistic pulmonary infections. The patient suffered from three episodes of clinical deterioration. In this case report, the patient's clinical deterioration and chest CT involvement were conspicuously associated with a high coronal viral load.

PCR‐based laboratory tests cannot differentiate infectious virus from non‐hazardous RNA of the same virus but still remain an interesting way of confirmation of viral infection.^4^ While there is much bulk of studies investigating COVID‐19 reinfection, known as subsequent infection after 4 weeks of previous infection clearance, and reactivation, known as subsequent infection within the next 4 weeks after previous infection clearance,^5^, ^6^ to the extent of our knowledge, there are few studies investigating prolonged COVID‐19 viral shedding. Although it is believed that median viral shedding is 20 days,^7^ it is discussed that in patients with underlying disorders, the median duration of SARS‐CoV‐2 RNA shedding was 53 days, and the maximum was 83 days.^3^

Reviewing the literature, it seems some COVID‐19 patients with concurrent lymphoma failed to remove the virus thoroughly, despite providing appropriate treatment regimens. Accordingly, in this study, we concluded that due to underlying immunological disorder, the immune system could never remove the virus thoroughly, despite proper antiviral prescription; hence, death was an inevitable outcome of multiple viral reactivations.

Moreover, based on the decreased cyclic threshold during clinical deterioration, it is believed that viral reactivation was the responsible agent for clinical exacerbation. Eventually, we believe that a disturbance in viral clearance, especially in immunological suppressed conditions such as lymphoma, inevitably leads to viral replication. Further studies are needed to investigate the association between the immunological signaling pathway disturbance and COVID‐19 clinical manifestation.

## AUTHOR CONTRIBUTIONS

Z.S was the head manager of the team. S.J helped in patient management and manuscript drafting. A.Kh contributed to manuscript drafting, reviewing, and submission. The authors declare that none of the authors listed in the manuscript are employed by a government agency and have no other function other than research and education. The authors are submitting this manuscript as an official representative.

## CONFLICT OF INTEREST

The authors declare that they have no conflicting interests.

## ETHICAL APPROVAL

Written informed consent was obtained from the patient's next of kin to publish this report in accordance with the journal's patient consent policy. All ethical and moral issues have been considered in this study.

## CONSENT

The consent form was obtained from the patient's first‐degree relatives after his death.

## Data Availability

All essential data have been included in this manuscript.
